# Syk‐dependent glycolytic reprogramming in dendritic cells regulates IL‐1β production to β‐glucan ligands in a TLR‐independent manner

**DOI:** 10.1002/JLB.3A0819-207RR

**Published:** 2019-09-11

**Authors:** Phyu M. Thwe, Daniel I. Fritz, Julia P. Snyder, Portia R. Smith, Kylie D. Curtis, Alexandra O'Donnell, Nicholas A. Galasso, Leslie A. Sepaniac, Benjamin J. Adamik, Laura R. Hoyt, Princess D. Rodriguez, Tyler C. Hogan, Andrea F. Schmidt, Matthew E. Poynter, Eyal Amiel

**Affiliations:** ^1^ Cellular, Molecular, and Biomedical (CMB) Sciences Graduate Program University of Vermont Burlington Vermont USA; ^2^ University of Vermont Burlington Vermont USA; ^3^ Vermont Lung Center Division of Pulmonary Disease and Critical Care Department of Medicine University of Vermont Burlington Vermont USA; ^4^ Department of Biomedical and Health Sciences University of Vermont Burlington Vermont USA

**Keywords:** dectin‐1/2, dendritic cells (DCs), glycolysis, inflammasome, NOD‐, LRR‐ and pyrin domain‐containing protein 3 (NLRP3), oxidative phosphorylation (OXPHOS), pattern recognition receptor (PRR), spleen tyrosine kinase (Syk), toll‐like receptor (TLR)

## Abstract

Dendritic cells (DCs) activated via TLR ligation experience metabolic reprogramming, in which the cells are heavily dependent on glucose and glycolysis for the synthesis of molecular building blocks essential for maturation, cytokine production, and the ability to stimulate T cells. Although the TLR‐driven metabolic reprogramming events are well documented, fungal‐mediated metabolic regulation via C‐type lectin receptors such as Dectin‐1 and Dectin‐2 is not clearly understood. Here, we show that activation of DCs with fungal‐associated β‐glucan ligands induces acute glycolytic reprogramming that supports the production of IL‐1β and its secretion subsequent to NOD‐, LRR‐ and pyrin domain‐containing protein 3 (NLRP3) inflammasome activation. This acute glycolytic induction in response to β‐glucan ligands requires spleen tyrosine kinase signaling in a TLR‐independent manner, suggesting now that different classes of innate immune receptors functionally induce conserved metabolic responses to support immune cell activation. These studies provide new insight into the complexities of metabolic regulation of DCs immune effector function regarding cellular activation associated with protection against fungal microbes.

## Graphical abstract

Fungal‐associated Beta‐glucan ligands induce acute glycolytic reprogramming in DCs in a Syk‐dependent TLR‐independent manner that contributes to IL‐1beta production.

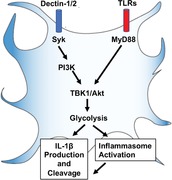

## 1 INTRODUCTION

Dendritic cells (DCs) are professional antigen‐presenting cells of the innate immune system. They play a critical role in initiating both innate and adaptive immune responses by serving as a bridge between these two systems.^1^, ^2^ They express a vast array of pattern recognition receptors (PRRs), such as TLRs, C‐type lectin receptors (CLRs), and mannose receptors.^3^, ^4^ Recognition of pathogen‐associated stimuli through these receptors induces DCs to up‐regulate transcription and translation of genes and proteins associated with their immune effector function, such as surface expression of co‐stimulatory molecules and MHC class II, and production of proinflammatory cytokines and chemokines. Subsequent to activation, DCs migrate to secondary lymphoid organs and present antigens to T cells, thereby initiating the adaptive immune responses.^1^

Among the PRRs, CLRs can recognize various lengths of carbohydrates through binding of polysaccharide moieties in C‐type lectin domains of the receptors. Dectin‐1 is a CLR expressed primarily on myeloid cells, such as macrophages and DCs, and on a small subset of lymphocytes.^5^, ^6^ Among the complex multilayers of fungal cell walls, O‐linked mannosylated proteins and mannans in the thin outermost layer are recognized by TLRs, whereas β‐glucan molecules, the most abundant layer of the fungal cell wall, are primarily recognized by Dectin‐1 and Dectin‐2 receptors.^7^ Innate immune responses to fungal infection are orchestrated by the cooperative recognition of pathogen‐associated molecular patterns (PAMPs) on the fungal cell wall via TLRs and CLRs. Previous studies have reported that fungal recognition by immune cells induces their activation via stimulation of TLR2 and/or TLR4, in addition to Dectin molecules.^8^

β‐glucans capable of stimulating Dectin‐1 and ‐2 exist in β1,3‐ and β1,6‐ linkages. These Dectin agonists are commercially available from the preparation of yeast *Saccromyces cervisiae* or *Candida albicans* and have been shown to elicit proinflammatory immune responses in macrophages and DCs.^5^, ^9^, ^10^ Although innate immune responses to whole yeast cell‐wall preparations are known to be mediated by both Dectin molecules and TLR‐2 receptors,^11^, ^12^ studies have shown that modified yeast‐derived molecules such as whole glucan particles (WGP) or depleted zymosan (ZD), which is deprived of TLR stimulatory capacity by treatment of zymosan (Zy) with hot alkali, produce Dectin‐specific inflammatory responses, such as reactive oxygen species (ROS) and cytokine production in a TLR‐independent manner.^13^, ^14^ Furthermore, Dectin‐specific responses are nonredundant with TLR‐mediated innate immune responses, as Dectin‐1‐deficient mice display impaired immune responses against *C. albicans*, resulting in compromised resistance to fungal infection.^10^

Stimulation of PRRs by PAMPs, such as microbial pathogens, and/or danger‐associated molecular patterns (DAMPs), such as ATP and uric acid crystals, results in release of proinflammatory cytokines by DCs and macrophages. Among these cytokines, IL‐1β plays a crucial role in both local and systemic inflammation as one of the earliest inflammatory mediators released by activated innate immune cells. The secretion of biologically active IL‐1β is mediated by the cleavage of pro‐IL‐1β by active caspase‐1, and is tightly controlled by 2 distinct cellular signals. PRR activation, which induces the transcription and translation of immunologically inactive pro‐IL‐1β serves as a first signal, which is followed by the second signal that causes the cleavage of pro‐IL‐1β by active caspase‐1. Different forms of a heterogeneous group of multiprotein complexes, termed inflammasomes, are responsible for activating caspase‐1. These inflammasome complexes consist of sensor proteins, such as nucleotide‐binding oligomerization domain like receptor family proteins (NLRs) that can detect PAMPs and/or DAMPs, an adaptor protein apoptosis‐related speck‐like protein (ASC), a pyrin or caspase recruitment domain, and effector caspase‐1. Inflammasome activation gives rise to autocatalysis and activation of caspase‐1, which then exerts a catalytic activity on pro IL‐1β.^15^, ^16^, ^17^ Among different types of NLR inflammasomes, activation of the NLRP3 inflammasome is most abundantly studied in the field of myeloid immune cells in response to various microbial pathogens, including fungi, and plays a documented downstream role in Dectin‐1‐initiated innate immune responses.^18^, ^19^, ^20^ Recent studies have reported that NLRP3 inflammasomes are required for immune responses against *C. albicans* mediated by both Dectin‐1 and TLR2; however, IL‐1β secretion is completely ablated in Dectin‐1 deficient mice compared to that in TLR2 knockout mice.^19^, ^21^, ^22^ Although the immune effector responses in these studies have been reported to require both TLR2 and Dectin receptors to fight fungal infections, Dectin‐1‐mediated effects on downstream NLRP3 inflammasome activation are much more pronounced.^21^

Activation through TLRs causes DCs to exhibit rapid changes in cellular metabolism characterized most prominently by increased rates of aerobic glycolysis. TLR‐driven glycolytic reprogramming critically supports the survival and immune function of DCs by satisfying both the energetic and nutrient substrate requirements associated with the rapid anabolic synthesis of proteins and effector molecules during DC activation.^23^, ^24^, ^25^, ^26^ Pharmacologic or genetic approaches to inhibit the induction of aerobic glycolysis during DC activation result in attenuation of DC maturation and lead to subsequent adverse effects on DC effector functions, including inflammatory cytokine secretion, antigen presentation, and T cell stimulation.^25^, ^26^, ^27^ A prominent recent study has demonstrated that monocytes challenged with a β‐glucan cell wall component of *C. albicans* up‐regulate aerobic glycolysis, albeit the underlying mechanism for this process is epigenetically regulated after a long‐term fungal challenge.^28^ Although activation of the NLRP3 inflammasome in macrophages is supported by aerobic glycolysis,^18^ and inhibition of glycolysis attenuates IL‐1β release and inflammasome activation in macrophages,^18^, ^29^ little is known about the how Dectin‐specific acute glycolytic induction contributes to inflammasome activation in DCs. Here, we report that DC stimulation with Dectin‐specific ligands drives aerobic glycolysis to support inflammasome‐dependent IL‐1β release, in a TLR‐independent manner.

Upon activation of Dectin‐1, the downstream adaptor motif of its cytoplasmic tail, which is similar to immunoreceptor tyrosine‐based activation motifs (ITAMs), serves as a docking site for spleen tyrosine kinase (Syk).^30^ Syk signaling plays an important role in lymphocyte proliferation and survival. In macrophages and DCs, phosphorylation of Syk induces inflammatory responses such as cytokine secretion,^31^ ROS production, and NLRP3 inflammasome activation.^30^, ^32^ The PI3K/Akt axis regulates the proliferation, survival, and metabolic activities in many cells.^33^, ^34^, ^35^ We have shown the activation of PI3K and Akt signal transduction during the TLR‐driven glycolytic burst in DCs.^24^ However, whether the PI3K/Akt axis is associated with Syk activity in Dectin‐stimulated DCs is not understood. Additionally, TLR signaling has been shown to depend on MyD88,^36^, ^37^ which is an essential signaling adaptor molecule in most TLR signaling pathways. Although some reports indicate the involvement of MyD88 in the immune responses to fungal pathogens,^31^, ^38^, ^39^ the role of MyD88 in Dectin receptor activation is controversial.^38^, ^40^ In this report, we demonstrate that NLRP3‐mediated IL‐1β secretion from cells primed by a Dectin‐specific ligand is facilitated by a Syk‐dependent and PI3K/Akt‐dependent glycolytic reprogramming that is independent of TLR‐MyD88.

Although the importance of glycolytic reprogramming for DC effector function has been extensively studied in TLR‐driven activation, DC metabolic regulation in response to fungal pathogens is understudied. In this study, we show that the Dectin‐mediated glycolytic burst is regulated by Syk activation in a TLR‐independent manner. Syk inhibition abolished the Dectin‐ driven glycolytic burst and downstream IL‐1β activation, while sparing MyD88‐dependent TLR stimulation. Collectively, our study suggests that DCs engage in a Syk‐dependent signaling mechanism responsible for driving the Dectin‐specific glycolytic burst in support of IL‐1β production and release. These findings show that CLRs, in addition to TLRs, are sufficient for driving early glycolytic reprogramming in DCs, and that this activity is important for sustaining the early inflammatory responses to fungal‐associated ligands.

## 2 METHODS

### 2.1 Mice and reagents

C57BL/6J, TLR2^−/−^, and MyD88^−/−^ mice were purchased from Jackson Laboratory (Bar Harbor, ME) and bred in‐house and maintained at the University of Vermont animals care facility under protocols approved by Institutional Animal Care and Use Committee. Endotoxin free LPS (*Escherichia coli* serotype O), Pam2Csk4, Zy, ZD, Curdlan, and WGP were purchased from Invivogen (San Diego, CA). 2‐deoxy‐glucose (2DG) was purchased from Sigma (St. Louis, MO). Antibodies for flow cytometry: 7‐Aminoactinomycin D (7‐AAD), anti‐CD11c (clone N418), anti‐CD86 (clone GL‐1), and anti‐CD40 (clone 3/23) antibodies were purchased from BD Biosciences (San Jose, CA) and Biolegend (San Diego, CA). For Western blot analysis, all antibodies were from Cell Signaling (Danvers, MA) (phosph‐Akt T308 [clone D25E6]), pan‐Akt (clone C67E7), cleaved caspase‐1 (clone E2G2I), and cleaved IL‐1β (clone E7V2A) except for β‐actin (clone 643802), which was purchased from Biolegend. PI3K (20 uM), Syk (10 uM), and TBK1 (5 uM) pharmacologic inhibitors (LY294002, PRT062607, and BX‐795 respectively) were purchased from Selleckchem (Houston, TX).

### 2.2 Mouse DC culture and activation

Bone marrow‐derived DCs (BMDCs) were generated as described in Lutz et al.^41^ Briefly, bone marrow hematopoietic cells were differentiated in GM‐CSF (20ng/uL; Shenandoah Biotechnology Inc., Warwick, PA) in complete DC medium (CDCM), comprising RPMI 1640, 10% FCS, 2 mM L‐glutamine, 1IU/mL Pen‐Strep, 1 mM beta‐mercaptoethanol, for 7 d. For 5 mM glucose media, glucose‐free RPMI is used as a base media in addition to dialyzed FCS, and glucose added to 5 mM concentration. On day 7, DCs were washed in CDCM and cultured at 2 × 10^5^ cells per 200 µL of media. For intracellular cytokine staining, cells were activated for a total of 4 h with an addition of Golgi plug (Biolegend) after the first hour of stimulation.

### 2.3 Western blot analysis

DCs were lysed using lysis buffer with Pierce protease and phosphatase inhibitors. For cell lysate analysis, protein levels were quantified using the Pierce BCA Assay kit (Thermo Fisher, Waltham, MA) and normalized to 20–30 µg of total protein (depending on the individual blot) prior to running on 12% SDS‐PAGE gels and subsequent transfer to nitrocellulose membranes. For cell supernatant analysis, 2–4 million cells were stimulated in 2 mL of media and supernatant were concentrated 10‐fold using StrataClean Resin (Agilent, Santa Clara, CA) to nonspecifically concentrate all proteins in the supernatant. Cleaved caspase‐1 and cleaved IL‐1β blots were performed on these concentrated supernatant preparations.

### 2.4 Metabolism assays

Extracellular acidification rate (ECAR) and oxygen consumption rate (OCR) were measured with Metabolic Flux Analyzer (Agilent, Santa Clara, CA, USA; 24XP and/or 96XP).

### 2.5 Flow cytometry and cytokine measurements

Above mentioned antibodies were used for flow cytometry. For intracellular staining of TNF‐α and IL‐12 (Biolegend, San Diego, CA), cells were fixed in 4% paraformaldehyde, permeabilized in 0.2% saponin, and stained with the antibodies in FACS buffer (1% BSA in PBS). Samples were analyzed on a BD Biosciences LSRII flow cytometer. For cytokine levels, supernatants were collected as indicated time points and measured by Duo‐set ELISA kits (R&D Systems, Minneapolis, MN).

### 2.6 Quantitative real‐time PCR of Il‐1β expression

RNA was isolated with an RNAeasy Kit (Qiagen, Germantown, MD) and cDNA was synthesized with an iScript cDNA Synthesis Kit (Biorad, Hercules, CA). *Il‐1β* Taqman primer probes (Applied Bioscience System, Foster City, CA) and AB7500 sequence detection system or QuantStudio 3.0 were used for relative mRNA expression. mRNA relative quantitative values were calculated based on 2(‐ΔΔCT) and were normalized to untreated DCs with β‐actin used as a housekeeping gene control.

### 2.7 Statistical analysis

Data were analyzed with GraphPad Prism software (version 6.0). Samples were analyzed using paired *t*‐test, 1‐way and 2‐way ANOVA. ANOVA tests were post‐calculated by Tukey's multiple comparison test. Results are means +sd or +sem, and statistical values are represented with an asterisk as significant when *P* values were below 0.05.

## 3 RESULTS

### 3.1 Dectin‐mediated activation drives glycolytic reprogramming

**Figure 1:**
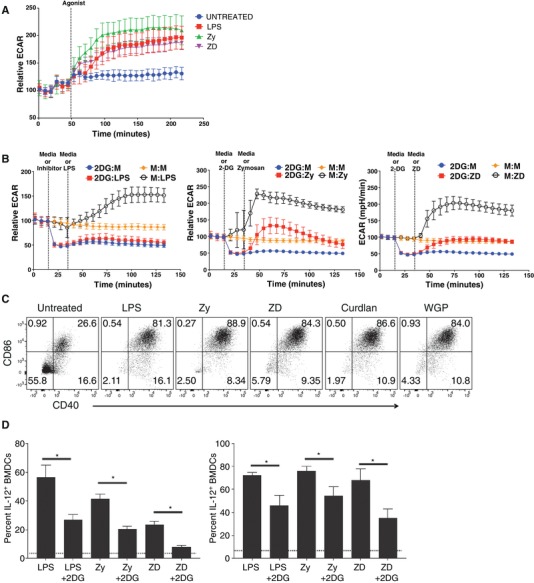
**Dectin‐mediated activation drives glycolytic reprogramming**. (**A**) Bone marrow‐derived dendritic cells (BMDCs) were stimulated with LPS (10 ng/mL), zymosan (Zy; 100 µg/mL), depleted zymosan (ZD; 100 µg/mL), and extracellular acidification rate (ECAR) is measured in real time using a Seahorse Extracellular Flux Analyzer (Agilent, Seahorse Biosciences). (**B**) Relative ECAR of BMDCs stimulated with 5 mM 2DG followed by media or corresponding ligands (LPS, Zy, or ZD). (**C**) Surface expression of CD40 and CD86 on BMDCs stimulated for 24 h with LPS, Zy, ZD, Curdlan, or whole glucan particles (WGP). (**D**) Intracellular cytokine staining of TNF and IL‐12 of BMDCs stimulated for 6 h with LPS, Zy, ZD in 5 mM glucose in the presence or absence of 2DG was performed. Percentage of cytokine‐positive cells is indicated. Bar graphs shows mean ± sd for 3 biological replicates; asterisk indicates significance of *P* < 0.05; and dotted line represents the mean background percentage for unstimulated cells. Data are representative of more than 3 independent experiments

In order to identify Dectin‐specific acute metabolic reprogramming in DCs and to determine the contribution of these metabolic changes to early immune responses by these cells, we utilized an array of agonistic ligands specific to TLRs alone (LPS), Dectin‐1/2 alone (ZD, Curdlan, WGP), or ligands that interact with both simultaneously (Zy). We first characterized the ability of these different agonists to induce acute glycolytic reprogramming, termed “glycolytic burst,” in GM‐CSF‐differentiated BMDCs by metabolic extracellular flux analysis (Agilent, Seahorse Biosciences). By stimulating the cells with the various agonists above, we were able to determine the ability of these receptors to mediate acute glycolysis induction. Consistent with our previous work in which TLR4 stimulation with LPS induced a rapid glycolytic burst, we observed a similar metabolic response to both Zy and ZD stimulation (Fig. 1A). 2DG, a synthetic molecule structurally similar to glucose that functions as an inhibitor of glycolysis, dramatically attenuated the glycolytic burst induced by all 3 ligands, suggesting that Dectin‐1 activation drives the aerobic glycolytic burst (Fig. 1B). To verify that each member of our agonist panel was able to induce DC maturation comparably, we next characterized the induction of surface co‐stimulatory molecule expression in BMDCs stimulated for 24 h with each ligand (Fig. 1C). Furthermore, we observed the involvement of glycolytic metabolism to support activation‐associated TNF‐α and IL‐12 production in response to each ligand (Fig. 1D). Consistent with our metabolic data (Fig. 1A and B), proper DC activation by all ligands, including Dectin‐specific ligand, ZD, requires glycolytic reprogramming (Fig. 1D).

### 3.2 Dectin‐dependent glycolytic burst and maturation requires Syk signaling

**Figure 2:**
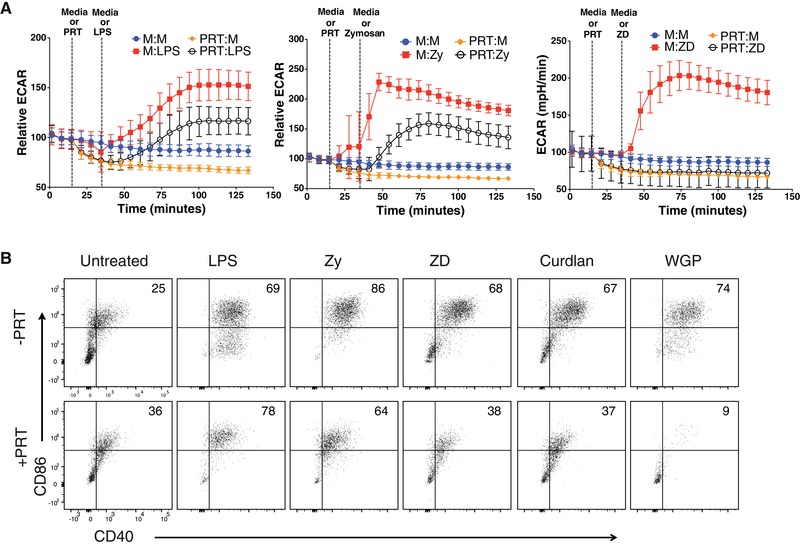
**Dectin‐dependent glycolytic burst and maturation requires Syk signaling**. (**A**) Real‐time Relative extracellular acidification rate (ECAR) of bone marrow‐derived dendritic cells (BMDCs) treated with media control (M) or Syk inhibitor (PRT) followed by stimulation with LPS, Zy (zymosan), or ZD (depleted zymosan). The legend indicates the treatment order at the 2 injection points, where “M:M” indicates media injection at both time points. (**B**) CD40 and CD86 surface expression of BMDCs stimulated for 24 h with LPS, Zy, ZD, Curdlan, or whole glucan particles (WGP) in the presence or absence of Syk inhibitor (PRT). Data are representative of more than 3 independent experiments

Dectin‐driven immune responses are documented to be mediated by both Syk‐dependent and ‐independent mechanisms.^5^, ^42^ Based on this, we were interested in testing the requirement for Syk signaling in supporting Dectin‐mediated glycolytic burst and effector function in DCs. We examined acute glycolysis induction in BMDCs stimulated with TLR and/or Dectin ligands in the presence or absence of the Syk inhibitor, PRT. Interestingly, PRT completely ablated glycolysis induction in the cells stimulated by the Dectin‐specific ligand, ZD, whereas only a partial inhibition was observed for the TLR‐specific agonist LPS and dual agonist Zy (Fig. 2A). The partial effect of Syk inhibition on LPS‐mediated glycolytic reprogramming is consistent with reports that Syk constitutively binds to the cytoplasmic tail of TLR4 and is known to play a role in TLR4‐mediated signaling.^43^ Up‐regulated surface expression of the co‐stimulatory molecules CD40 and CD86 in response to Dectin‐specific ligands (ZD, Curdlan, WGP) was strongly attenuated by Syk inhibition, whereas TLR‐driven maturation (LPS) was unimpaired, and dual‐agonist maturation (Zy) was only modestly impaired, by Syk inhibition (Fig. 2B). These data indicate that Syk‐dependent signals are sufficient to regulate Dectin‐mediated glycolytic reprogramming and maturation in DCs.

### 3.3 Dectin‐mediated metabolic reprogramming is independent of TLR/MyD88 signals

**Figure 3:**
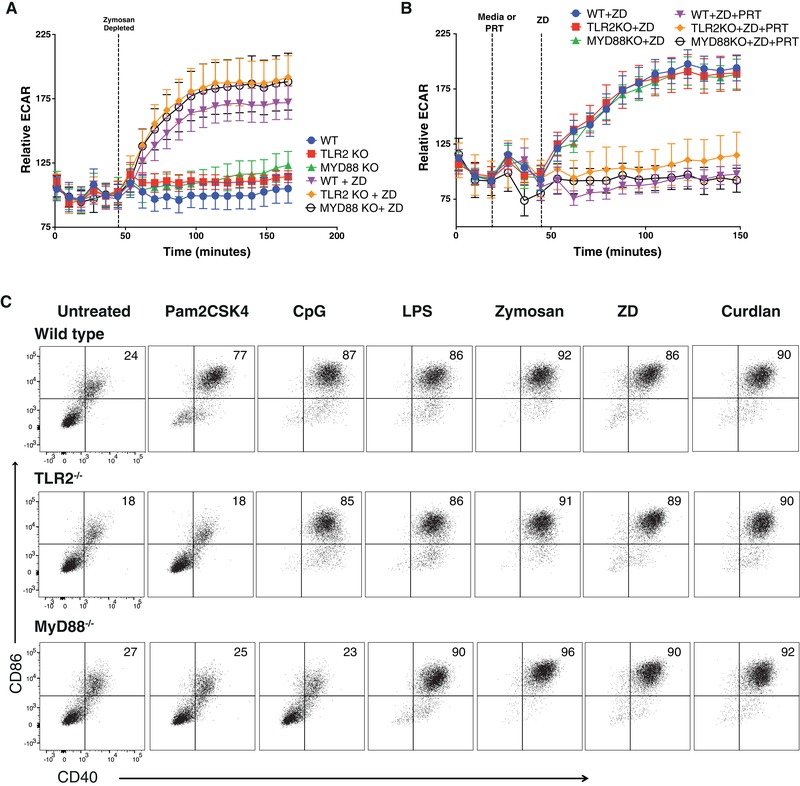
**Dectin‐mediated glycolytic reprogramming is independent of TLR2/MyD88 signals**. (**A**) Relative extracellular acidification rate (ECAR) of bone marrow‐derived dendritic cells (BMDCs) from wild‐type (WT), TLR2**^−/−^**, and MyD88**^−/−^** mice stimulated in real time response to ZD. (**B**) Relative ECAR of BMDCs from WT, TLR2**^−/−^**, and MyD88**^−/−^** mice stimulated in real time response to Syk inhibitor (PRT) followed by ZD. (**C**) CD40 and CD86 surface expression of BMDCs WT, TLR2**^−/−^**, and MyD88**^−/−^** stimulated for 24 h with Pam2CSK4 (TLR2 ligand), CpG (TLR9 ligand), LPS (TLR4 ligand), Zy (TLR2 and Dectin‐1 ligand), ZD, or Curdlan (Dectin‐1 ligands). Data are representative of more than 3 independent experiments

In order to confirm that Dectin‐specific metabolic reprogramming and activation was independent of TLR‐signaling, we stimulated DCs from TLR2^−/−^ and MyD88^−/−^ mice and assessed their ability to induce acute glycolytic burst in response to Dectin‐specific activation (Fig. 3A). DCs exhibited comparable levels of glycolysis induction in response to ZD even in the absence of TLR2 or MyD88 expression (Fig. 3A). We next activated wild‐type (WT), MyD88^−/−^, and TLR2^−/−^ BMDCs with ZD in the presence or absence of the Syk inhibitor PRT. Syk inhibition completely ablated Dectin‐1‐mediated glycolysis induction in all genotypes (Fig. 3B). Furthermore, TLR2^−/−^ BMDCs were unresponsive to canonic TLR2 agonist Pam2Csk4, and MyD88^−/−^ BMDCs did not mature in response to both TLR2 and TLR9 agonists as expected, whereas all genotypes matured properly in response to LPS (presumably via the MyD88‐independent TIR domain‐containing adaptor‐inducing interferon‐B (TRIF) and trif‐related adapter molecule (TRAM) signaling pathway) and Dectin‐specific ligands (Fig. 3C). These data confirm that Dectin/Syk mediated signals are sufficient to drive DC metabolic reprogramming even in the absence of the TLR2/MyD88 signaling axis that is also well documented to regulate innate immune responses to fungal‐associated ligands.

### 3.4 Dectin‐mediated glycolytic reprogramming requires a PI3K/TBK‐1/Akt signaling axis

**Figure 4:**
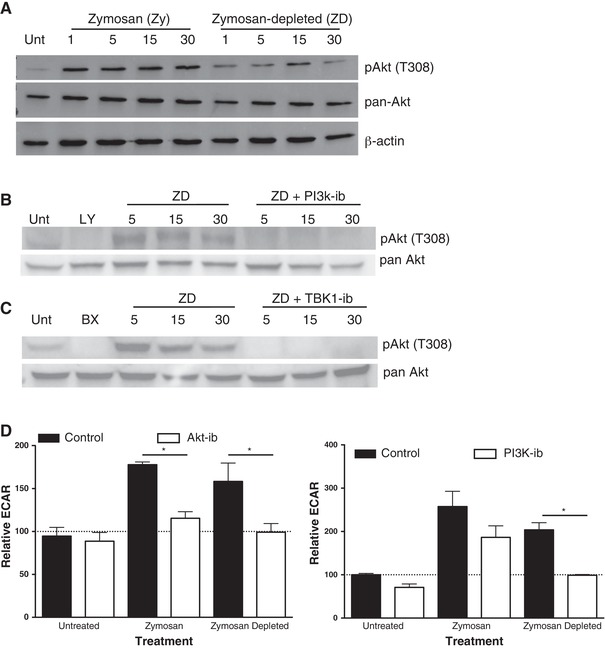
**Dectin‐mediated glycolytic reprogramming requires a PI3k/TBK1/Akt signaling axis**. (**A**) Protein expression of pAkt (T308), pan Akt, and β‐actin in BMDCs stimulated with Zy and ZD for 1, 5, 15, and 30 min. (**B**) Protein expression of pAkt (T308) and total Akt in BMDCs stimulated for 5, 15, and 30 min with ZD in the presence or absence of PI3K inhibitor (LY). (**C**) Protein expression of pAkt (T308) and total Akt in BMDCs stimulated with ZD in the presence or absence of TBK1 inhibitor (BX) for 5, 15, and 30 min. (**D**) Relative extracellular acidification rate (ECAR) of BMDCs stimulated in real time response to Zy and ZD in the presence or absence of PI3K or Akt inhibitors. Data represents *n* = 4; ^*^ *P* < 0.05 Student's *t* test

Syk signaling is known to play a crucial role in Dectin‐mediated innate immune responses.^22^, ^30^, ^31^, ^44^ In previously published work, we have identified that phosphorylation of Akt T308 is required for hexokinase association with the mitochondria and glycolytic reprogramming in TLR‐stimulated DCs.^24^ We thus hypothesized that similar signaling pathways may regulate acute metabolic responses to Dectin‐specific activation of DCs. To test this, we first examined expression of total and T308‐phosphorylated Akt in BMDCs stimulated with Zy or ZD (Fig. 4A). We observed that DC activation by both ligands induces the phosphorylation of Akt T308, suggesting that TLR and CLR signaling mechanisms likely converge to mediate glycolytic reprogramming (Fig. 4A). PI3K inhibition resulted in reduced phosphorylation of Akt T308 in response to ZD, suggesting that PI3K is involved in regulating Dectin‐dependent signaling that leads to Akt T308 phosphorylation (Fig. 4B). This is in contrast to previously published data showing that PI3K is not required for acute glycolytic reprogramming in response to TLR stimulation in DCs.^24^ In previous studies, we have shown that early TLR‐mediated glycolytic reprogramming in DCs is mediated by signaling through TBK1.^24^ Consistent with these findings, inhibition of TBK1 completely ablated the phosphorylation of Akt T308 in ZD activation (Fig. 4C), suggesting that TLR and Dectin‐dependent glycolysis induction pathways converge at this point. Consistent with these data, Akt inhibition attenuated acute glycolytic reprogramming in both Zy‐ and ZD‐stimulated DCs (Fig. 4D, left), whereas PI3K inhibition significantly impacted glycolysis induction only in ZD‐stimulated cells (Fig. 4D, right). In TLR‐stimulated DCs, TBK1 phosphorylation of Akt has been demonstrated to regulate acute glycolytic burst independent of PI3K signaling, whereas the PI3K/Akt axis plays a major role in maintaining long‐term glycolytic activity in these cells.^24^, ^26^ In contrast to these findings for acute glycolytic induction to TLR‐mediated stimulation, our data suggest that Dectin‐dependent Akt T308 activation is mediated by both PI3K and TBK‐1.

### 3.5 Dectin‐mediated acute glycolytic reprogramming is required for IL‐1β secretion triggered by the NLRP3 activator nigericin

**Figure 5:**
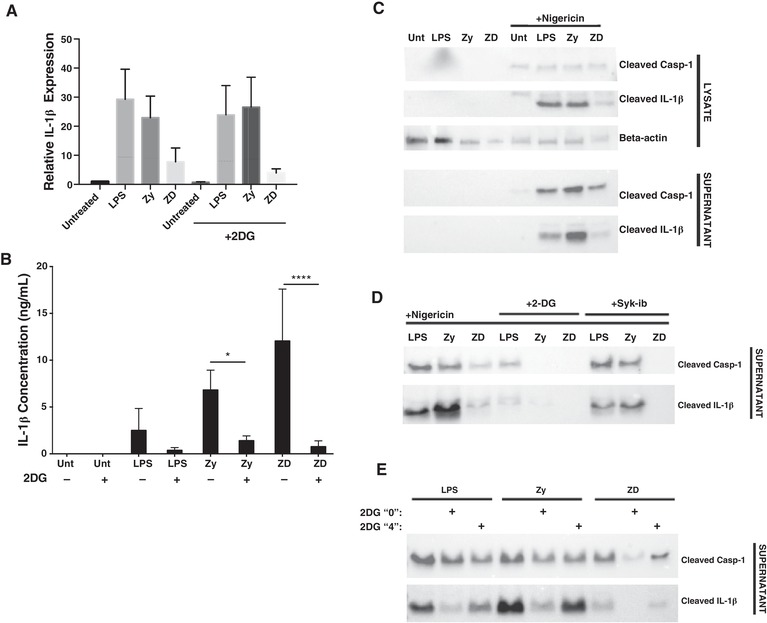
**Dectin‐mediated acute glycolytic reprogramming is required for NLRP3‐dependent IL‐1β secretion**. (**A**) Relative mRNA expression of IL‐1β in BMDCs stimulated for 6 h with LPS, Zy, or ZD in the presence or absence of 2DG, normalized to β‐actin expression. Data represents *n* = 4; ^*^ *P* < 0.05 ANOVA test. (**B**) ELISA determination of total (cleaved and uncleaved) secreted IL‐1β in dendritic cells stimulated for 4 h with indicated agonists and 1 h of nigericin. Data represents *n* = 4; ^*^ *P* < 0.05 ANOVA test. (**C**) Cleaved caspase‐1 and cleaved IL‐1β protein expression in cell lysates and concentrated culture supernatants from BMDCs stimulated with LPS, Zy, and ZD for 5 h with an addition of nigericin in the last hour. (**D**) Cleaved caspase‐1 and cleaved IL‐1β protein expression in BMDC concentrated culture supernatants stimulated with LPS, Zy, and ZD in the presence or absence of 2DG or PRT for 5 h with an addition of nigericin in the last hour. (**E**) Cleaved caspase‐1 and cleaved IL‐1β protein expression in BMDC concentrated culture supernatants stimulated with LPS, Zy, and ZD for 5 h with an addition of nigericin in the last hour in the presence or absence of 2DG throughout (2DG “0”) or 2DG at the time of nigericin addition (2DG “4”). Western blot data are representative of at least 3 independent experiments

In macrophages, glycolytic reprogramming has been demonstrated to drive NLRP3 inflammasome activation initiated by 2 signal components, LPS as a first signal and ATP or nigericin as a second.^18^ Whereas Cheng et al. have shown that human monocytes challenged with β‐glucan components of *C. albicans* up‐regulate aerobic glycolysis, these studies emphasized the long‐term effect of β‐glucan‐treated monocytes and the metabolic changes described in these cells have been documented to be regulated by epigenetic changes.^28^, ^45^ However, the requirement for Dectin‐specific acute metabolic regulation for NLRP3 mediated IL‐1β secretion has not been explicitly characterized in DCs. We hypothesized that the aerobic glycolysis observed in Dectin‐activated BMDCs (Figs. 1 and 2) is required to support IL‐1β production and secretion triggered by the NLRP3 inflammasome in a Syk‐dependent manner. To test this, we activated BMDCs with LPS, Zy, and ZD in the presence or absence of 2DG for 6 h and examined the transcriptional expression of IL‐1β (Fig. 5A). mRNA expression of IL‐1β did not change regardless of glycolysis inhibition, indicating that IL‐1β production is not regulated by glycolysis at the transcriptional level, a finding consistent with the lack of a glycolytic requirement for transcription of inflammatory genes in response to TLR stimulation of DCs.^24^ However, using an ELISA for secreted IL‐1β, we found that IL‐1β production and secretion was significantly reduced in 2DG‐inhibited DCs at 5 h post‐activation (Fig. 5B). This ELISA assay did not discriminate between pro‐ and cleaved IL‐1β, and so does not directly assess NLRP3 inflammasome activation; rather, it depicts an impact on IL‐1β protein translation that is consistent with the attenuation of cytokine protein production seen in TLR‐activated 2DG‐inhibited DCs.^24^ To more directly address the impact of glycolysis on NLRP3‐dependent activity, the levels of both cleaved caspase‐1 and cleaved IL‐1β were detected in both cellular lysates and culture supernatants (Fig. 5C). Cleaved caspase‐1 and cleaved IL‐1β were both readily detected in lysates and supernatants of cells stimulated with LPS, Zy, or ZD in the presence of the inflammasome complex activator nigericin, with ZD‐stimulated cells showing the lowest levels of these cleaved molecules (Fig. 5C). Cleaved caspase‐1 and cleaved IL‐1β levels in BMDCs stimulated with the TLR/CLR agonists in the presence of nigericin were significantly attenuated by 2DG‐mediated glycolysis inhibition (Fig. 5D). It is worth noting that in our hands, cleaved IL‐1β levels were consistently more sensitive to 2DG inhibition than cleaved caspase‐1, and that the effect of 2DG on cleaved caspase‐1 was fairly variable ranging from near‐complete inhibition (Fig. 5D) to moderate (Fig. 5E). Syk inhibition completely ablated cleaved caspase‐1 and cleaved IL‐1β levels in ZD‐stimulated cells, and only partially attenuated these responses in LPS or Zy‐stimulated DCs, consistent with the differential dependence on Syk signaling for these ligands shown in Figures 2 and 5D. Together, these data support the requirement of Syk‐dependent signals for both glycolytic induction and the cleavage of IL‐1β prior to its secretion in these cells (Figs. 1B and 5). To try to distinguish between glycolysis‐dependent protein translation and glycolysis‐dependent NLRP3 inflammasome‐mediated IL‐1β secretion, we performed a staggered 2DG inhibition study in which we compared 2DG addition at the start of TLR/CLR agonist treatment (Fig. 5E; 2DG “0” group) with addition of 2DG only for the final hour during nigericin treatment (Fig. 5E; 2DG “4” group). By adding 2DG for only the final hour during nigericin treatment, we expected to determine the requirement of glycolysis during nigericin‐dependent NLRP3 inflammasome formation but independent of the 4 h “priming” step mediated by transcription/translation events downstream of our experimental agonists (Fig. 5E). Extracellular cleaved caspase‐1 levels were modestly impacted by 2DG treatment at either time point, with ZD‐stimulated cells showing the highest sensitivity to glycolysis inhibition (Fig. 5E). In addition, cleaved IL‐1β production and secretion was attenuated for all ligands by 2DG inhibition at both “0” and “4” h time points, with more dramatic impact in the 2DG “0” group and a more modest impact for the 2DG “4” group (Fig. 5E). These data suggest that Dectin‐mediated and Syk‐dependent IL‐1β activity is regulated at both the translational level and inflammasome‐dependent secretion level by glucose metabolism (Fig. 5D and E).

## 4 DISCUSSION

Stimulation of myeloid immune cells with fungal ligands induces metabolic reprogramming.^28^, ^46^ Several studies have also shown that activation (of immune cells) by fungal pathogens causes inflammasome formation^17^, ^32^, ^44^, ^47^ and induces the Syk‐kinase signaling pathway.^22^, ^30^, ^31^, ^44^ Although these studies add significant value to the understanding of host immune responses against fungal pathogens, an integrated understanding of fungal‐mediated acute metabolic changes in the regulation of specific immune outcomes in DCs, such as inflammasome‐dependent cytokine responses, has not been previously described. In this study, we demonstrate that the Dectin‐specific activation of DCs induces glycolysis‐dependent IL‐1β production via the Syk‐mediated signal transduction pathway, which drives activation of the PI3K/TBK1/Akt axis. By activating BMDCs with Dectin‐specific ligands, we have been able to discriminate between Dectin‐associated glycolytic burst and effector responses from TLR‐mediated effects. We show that β‐glucan ligands induce rapid glycolytic reprogramming in DCs, and DC maturation by Dectin‐specific ligands is dependent on glucose in a TLR2‐ and MyD88‐independent manner.

In this study, we have taken a reductionist approach using purified ligands to interrogate the contribution of specific receptors to anti‐fungal metabolic responses. However, the composition of fungal cell walls is complex, and understanding the contribution of a single receptor to innate immune cell metabolism is a challenge. Recent work by Netea et al. has elegantly shown that activation of monocytes with hyphae or yeast cells results in a differential utilization of metabolic pathways, in which yeast‐activated immune cells are dependent on both glycolysis and TCA metabolism, whereas hyphae activation induces the cells to rely solely on glycolysis.^46^ Earlier studies have reported that *C. albicans* hyphae is primarily recognized via Dectin‐2 receptors,^48^ suggesting that metabolic responses to nuanced components of fungal cell walls by different PRRs could initiate utilization of distinct metabolic pathways. It is worth noting that in a true pathophysiologic context, TLR and CLR receptors are simultaneously engaged by innate immune cells; nevertheless, deficiencies in Dectin molecules alone can lead to increased infection susceptibility, demonstrating that these pathways are not entirely redundant.^19^, ^21^, ^22^ Furthermore, we think that the study presented here demonstrates an interesting evolutionary convergence between TLR and CLR innate immune signaling pathways, whereby acute glycolysis induction is a requirement for proper downstream effector functions mediated by both families of receptors.

The TLR‐driven early glycolytic reprogramming is mediated by Akt‐dependent TBK1/IKKε signaling,^24^ and a long‐term commitment to aerobic glycolysis is facilitated by PI3K/Akt/mTOR signaling.^25^ Whereas MyD88, an upstream adaptor molecule of TLR‐mediated signaling pathways, is central to TLR‐driven effector responses, conflicting reports exist regarding the role of MyD88 in non‐TLR signaling pathways such as Dectin‐1‐driven responses.^22^, ^38^ In the present study, we examined if MyD88 is involved in Dectin‐specific metabolic regulation of DCs. We found that the Dectin‐dependent ligand, ZD, specifically triggers rapid glycolytic burst independent of TLR2 or MyD88. These data indicate that Dectin‐1‐specific signals, via a Syk/PI3K/Akt signaling axis, are sufficient to initiate both DC glycolytic reprogramming and maturation in response to activation by fungal‐associated ligands. These findings are consistent with the recent work of Ding et al. in which they showed that PI3K/Akt signaling regulates the proliferation of cytotoxic T cells activated by human monocytes induced with Dectin‐specific β‐glucan molecules.^34^

The pioneering work of Sousa et al. highlighted that Syk plays a central role for cytokine production by Dectin‐1 activation.^31^ Syk phosphorylation is also crucial for Dectin‐1‐mediated inflammasome formation and IL‐1β production.^22^, ^32^ Although the TLR‐MyD88 axis is not essential for Dectin‐1‐mediated inflammasome formation in BMDCs, clear distinctions between Dectin‐1‐ and TLR‐mediated signaling components have not been fully established.^22^ This could be potentially due to the complex nature of fungal cell wall composition and the challenge of experimentally isolated single receptor‐specific responses. Here, we show that Dectin‐1/2 activation of BMDCs requires Syk signaling for glycolysis induction. Interestingly, we also showed that Dectin‐mediated Syk‐dependent IL‐1β production in BMDCs is heavily reliant on glucose and glycolytic reprogramming.

Glucose metabolism in TLR‐mediated activation has been widely studied in DCs and innate myeloid immune cells alike,^49^, ^50^ whereas few studies delineate the glycolytic requirement during immune cell activation via CLRs such as Dectin‐1/2. This work helps illustrate that glucose metabolism plays a central role in DC activation via distinct families of PRRs and that the up‐regulation of glycolysis is a classic metabolic hallmark in both TLR and CLR‐mediated activation. Interestingly, recent studies suggest that there may be nuanced differences in cellular metabolic responses to distinct types of microbial‐associated ligands. For instance, activation of human monocytes with Pam3Csk4 and LPS induces differential metabolic responses, in which Pam3Csk4 up‐regulates both glycolysis and oxidative phosphorylation (OXPHOS), whereas LPS induces aerobic glycolysis but not OXPHOS.^51^ Differential functional outcomes could also arise from cellular participation in divergent glucose metabolism. In the example of monocytes stimulated with TLR2 ligands, phagocytic activity of these cells is preferentially supported by OXPHOS whereas cytokine production is facilitated by glycolysis and OXPHOS.^51^ In parallel to TLR‐mediated metabolic changes, recent work has highlighted the differential metabolic outcomes from dimorphic stages of *C. albicans*, leading to diverse functional responses.^46^ Of note, since we have recently shown that TLR‐mediated DC activation relies on alternative metabolic pathways such as glycogen metabolism,^52^ it is tempting to hypothesize that Dectin‐1 mediated responses may participate in similar aspects of activation‐associated glucose metabolism.

In summary, our findings identify Dectin‐specific acute glycolytic reprogramming events that support DC effector responses to fungal‐associated β‐glucan ligands. However, the complex and redundant nature of the metabolic networks demands additional work at an organism level to better understand the regulatory mechanisms implicated in particular anti‐fungal immune responses. With a rising interest in metabolic regulation and consequent immune outcomes from various pathogenic stimuli, our work adds to an increasingly nuanced understanding of metabolic regulation of DCs in response to different microbial stimuli. These studies, as well as those by others in the field, underscore the need for additional research to elucidate the complexity of metabolic regulation of innate immune cell responses to different pathogens.

## AUTHORSHIP

P.M.T., D.I.F., and E.A. wrote the original draft of the manuscript; J.P.S. and E.A. wrote the revised manuscript. M.E.P. assisted with conceptualization, interpretation, technical assistance, and editing of the work. P.M.T., D.I.F., J.P.S., P.R.S., K.D.C., A.OD., N.A.G., L.A.S., B.J.A., L.R.H., P.D.R., T.C.H., A.F.S., and E.A., all performed experiments, and gathered and interpreted data in support of this work. P.M.T. and D.I.F. contributed equally to this article.

## DISCLOSURES

The authors declare no conflicts of interest.
